# MicroRNAs as serum biomarkers in Becker muscular dystrophy

**DOI:** 10.1111/jcmm.17462

**Published:** 2022-07-26

**Authors:** Delia Gagliardi, Mafalda Rizzuti, Roberta Brusa, Michela Ripolone, Simona Zanotti, Elisa Minuti, Valeria Parente, Laura Dioni, Sara Cazzaniga, Paolo Bettica, Nereo Bresolin, Giacomo Pietro Comi, Stefania Corti, Francesca Magri, Daniele Velardo

**Affiliations:** ^1^ Neurology Unit IRCCS Foundation Ca’ Granda Ospedale Maggiore Policlinico Milan Italy; ^2^ Department of Pathophysiology and Transplantation, Dino Ferrari Center University of Milan Milan Italy; ^3^ Neuromuscular and Rare Diseases Unit IRCCS Foundation Ca’ Granda Ospedale Maggiore Policlinico Milan Italy; ^4^ EPIGET Lab, Unit of Occupational Medicine, Department of Clinical Sciences and Community Health, IRCCS Ca’ Granda Foundation Ospedale Maggiore Policlinico University of Milan Milan Italy; ^5^ Italfarmaco SpA Milan Italy

**Keywords:** Becker muscular dystrophy, biomarkers, BMD, miR‐133b, miRNA, serum, skeletal muscle

## Abstract

Becker muscular dystrophy (BMD) is an X‐linked neuromuscular disorder due to mutation in the *DMD* gene, encoding dystrophin. Despite a wide clinical variability, BMD is characterized by progressive muscle degeneration and proximal muscle weakness. Interestingly, a dysregulated expression of muscle‐specific microRNAs (miRNAs), called myomirs, has been found in patients affected with muscular dystrophies, although few studies have been conducted in BMD. We analysed the serum expression levels of a subset of myomirs in a cohort of 29 ambulant individuals affected by BMD and further classified according to the degree of alterations at muscle biopsy and in 11 age‐matched healthy controls. We found a significant upregulation of serum miR‐1, miR‐133a, miR‐133b and miR‐206 in our cohort of BMD patients, supporting the role of these miRNAs in the pathophysiology of the disease, and we identified serum cut‐off levels discriminating patients from healthy controls, confiming the potential of circulating miRNAs as promising noninvasive biomarkers. Moreover, serum levels of miR‐133b were found to be associated with fibrosis at muscle biopsy and with patients' motor performances, suggesting that miR‐133b might be a useful prognostic marker for BMD patients. Taken together, our data showed that these serum myomirs may represent an effective tool that may support stratification of BMD patients, providing the opportunity of both monitoring disease progression and assessing the treatment efficacy in the context of clinical trials.

## 1 INTRODUCTION

Becker muscular dystrophy (BMD) is a neuromuscular disorder due to in‐frame mutations in the *DMD* gene, located on the X chromosome.^1^ This gene encodes for dystrophin, whose lack leads to structural damages and disruption of the membrane of skeletal muscles with consequent activation of inflammation and regeneration in the early phases of the disease and increase of connective and adipose tissue in later stages. In addition, other mechanisms such as neuronal nitric oxide synthase (nNOS) deficiency are involved in the pathogenesis of the disease.^2^

The pathological phenotype is characterized by a wide clinical variability, ranging from asymptomatic or slowly progressive proximal muscles weakness to more severe muscular involvement or isolated cardiomyopathy.^3^, ^4^ The disease course can be evaluated with both serologic and instrumental assessments. The creatine‐kinase (CK) activity is commonly dosed in muscular disorders, but it is not a specific disease marker, since it can vary according to loss of muscle mass, rhabdomyolysis, or intense muscular activity.^5^ The evaluation of muscle strength may be assessed using the Medical Research Council (MRC) scale,^6^ or through validated functional tests like Motor Function Measure (MFM) scale,^7^ North Star Ambulatory Assessment (NSAA) scale,^8^ the Six‐Minute‐Walking‐Test (6MWT)^9^ or temporized tests (10‐meters walk/run, 4‐stairs climb, rise from floor). Other clinical features, such as cardiomyopathy, arrhythmias and pulmonary function involvement, can be easily monitored with periodic electrocardiogram, echocardiogram and pulmonary function tests.

So far, no treatment is available for BMD, but experimental therapies could be available in the next few years. To date, the majority of clinical trials target patients with Duchenne muscular dystrophy (DMD), a more severe allelic disorder with infantile onset and absence of muscular dystrophin.^10^ In the DMD population, functional tests are generally used as outcome measures in clinical trials suggesting their relevance in both the evaluation and monitoring of patients affected by this disorder. Indeed, the outcome measures for BMD are currently the same used in other muscular dystrophies and include the functional scales previously cited with the 6MWT used as the most common clinical outcome measure to assess the efficacy of new potential therapeutic strategies^11^, ^12^ together with muscle biopsy, muscle MRI and evaluation of dystrophin expression. Nevertheless, considering the high heterogeneity and the slow progression of BMD, the majority of these outcome measures are not able to detect significant changes during a few years period, such as requested in clinical trials.^13^ Considering the clinical relevance of innovative therapies, there is an increasing need to identify specific disease biomarkers that can be used as complementary outcome measures, useful to define both the stage of the disease and prognosis during treatment, assessing the therapeutic effectiveness.

In recent years, microRNAs (miRNAs), small non‐coding RNA sequences, have been deeply studied to evaluate their role in the pathogenesis of multiple disorders as well as phenotype modifiers, biomarkers and therapeutic targets. Indeed, miRNAs can act as post‐transcriptional regulators, generally suppressing gene expression.^14^ Alteration of miRNAs expression has been linked to several neurological diseases, including neuromuscular disorders.^15^

MiRNAs selectively involved in muscular pathways, called myomirs or dystromirs (miR‐1, miR‐31, miR‐133a, miR‐133b, miR‐206),^16^ have been found upregulated in the serum of patients affected with muscular dystrophies, establishing serum microRNAs as promising and noninvasive biomarkers.^17^, ^18^ Among these miRNAs, miR‐31 is highly expressed in satellite cells, and it is involved in cellular regeneration and dystrophin expression inhibition.^19^ MiR‐1 and miR‐133a originate from the same transcript in the cardiac and skeletal muscles^20^: miR‐1 promotes myogenesis and terminal differentiation, while miR‐133a increases myoblasts proliferation.^21^ Similarly, miR‐206 and miR‐133b are codified by the same noncoding RNA: miR‐206 is specific for skeletal muscles, particularly activated satellite cells and proliferating myoblasts, and promotes injury‐related regeneration through differentiation and fusion of myotubes progenitors,^16^, ^22^ while miR‐133b is abundant in all except the cardiac muscles and has a role in early development of skeletal myocytes and neuromuscular synapses.^23^ In addition, miR‐133b seems to be involved in signalling pathways related to inflammation and fibrosis.^24^

Here, we aim to investigate myomirs serum levels in a cohort of BMD patients undergoing muscle biopsy and to compare them with those found in healthy subjects, in order to assess the presence of a myomir signature in BMD. Moreover, we aim at evaluating potential associations between serum myomirs with clinical and histological parameters and to assess the utility of serum miRNAs as prognostic tools. The identification of a unique diagnostic miRNA signature for BMD may lay the ground for their use as disease biomarkers useful for diagnosis, prognosis and monitoring of therapeutics effectiveness in clinical trials.

## 2 METHODS

### 2.1 Selection of participants

Patients enrolled in this study were adult males diagnosed with BMD by genetic testing, walking between 200 and 450 meters at 6MWT, without relevant cardiomyopathy (Ejection fraction [EF] > 50%) and with stable cardiologic therapy. Patients were recruited at the Neuromuscular and Rare Disease Unit/Neurology Unit of Fondazione IRCCS Ca′ Granda Ospedale Maggiore Policlinico of Milan. Male subjects with comparable ages and without neuromuscular disorders were enrolled as healthy controls.

### 2.2 Assessments

Clinical history, including age and site at onset, disease duration and current treatments, was registered for all the enrolled subjects. All patients underwent extensive clinical and neurological evaluations and functional tests, including timed function tests (4‐stairs climb, rise from floor, 10‐meters walk/run), MFM scale^25^ and 6MWT.^12^ MFM scale scores were registered for each item (D1: standing, position and transfers; D2: axial and proximal motor function; D3: distal motor function) and as a total.^25^ Blood was collected via a direct venous puncture for serum CK levels dosage in patients and for miRNA analysis in all the subjects. Both patients and controls did not perform physical exercise before blood collection. Patients underwent muscle magnetic resonance imaging (MRI), as already described by our group.^26^ Open muscle biopsy of the brachial biceps was performed in all patients and muscle tissue was analysed in the laboratory of the Neuromuscular and Rare Disease Unit.

### 2.3 Ethical statement

The study was conducted in accordance with the ethical standards of the Declaration of Helsinki and with national legislation and institutional guidelines. All subjects provided written informed consent approved by the local ethical committee for the collection, storage and analysis of blood samples. Moreover, patients provided written informed consent for muscle biopsy and collection of data of clinical evaluations.

### 2.4 Serum microRNAs analysis

Blood samples were centrifuged at 211 *g* for 10 min at 4°C. Circulating miRNAs were isolated from 300 μl of serum, using the NucleoSpin® miRNA plasma kit (Macherey Nagel). The total yield was assessed with Nanodrop, and the purity was estimated by the relative absorbance at A260/A280 ratio and A260/A230 ratio. Reverse transcription reactions were performed using the TaqMan® MicroRNA Reverse Transcription Kit (ThermoFisher SCIENTIFIC) and 4 ul of each RNA as template. Since preamplification is required to detect low‐expressing miRNAs, each RT product was preamplified using TaqMan® PreAmp Master Mix (ThermoFisher SCIENTIFIC). Real‐time PCR experiments were set up using the TaqMan® Universal Master Mix II No AmpErase® UNG (ThermoFisher SCIENTIFIC) and the appropriate 20× TaqMan® MicroRNA Assays (ThermoFisher SCIENTIFIC, probe ID available upon request). Plates were run on the 7900 Real Time PCR System (Applied Biosystems). The expression level of each miRNA was normalized to the average levels of hsa‐miR‐223^27^ and referred to control samples using the ΔCt method.

### 2.5 Muscle biopsy

Muscle biopsy samples from brachial biceps were immediately frozen in liquid nitrogen‐cooled 2‐methylbutane and then stored in liquid nitrogen. For histological analysis, 8 μm thick cryosections were routinely stained with haematoxylin and eosin. On each section, four randomly selected, nonoverlapping fields were photographed at 20× magnification, using an optical microscope (Leica DC200) equipped with a camera and IM50 image analysis software (Leica Microsystems). Each muscle biopsy was evaluated in a blinded manner by two expert operators. All morphometric analyses were performed using ImageJ 1.51j8 (https://imagej.nih.gov/ij/download.html) and LAS 4.9.0 (Leica Application Suite) software, consistent with the methods of Peverelli and colleagues.^28^ In each biopsy was evaluated the amount of muscle tissue (MFA) and the amount of fibroadipose tissue.

### 2.6 Western blot analysis

For immunodetection of endogenous dystrophin, a small fragment of muscle biopsy was homogenized in extraction buffer (4% SDS, urea 4 M). Lysate protein was loaded on 6% polyacrylamide‐SDS gel and transferred to a nitrocellulose membrane (Schleicher and Schuell). Membranes were probed with the dystrophin Rod domain mouse monoclonal antibody (NCL‐DYS1, dil. 1:200), from Novocastra Laboratories (Newcastle upon Tyne). Actinin (monoclonal antibody, 1:6000 Sigma Aldrich) was used as indicator of protein loaded. The membranes were incubated with rhodamine or fluorescein goat antimouse secondary antibodies (LI‐COR, Lincoln). Immunoreactive bands were visualized by ODYSSEY LI‐COR Model 2800 and quantitated densitometrically using Image J 1.46r software. Dystrophin band was normalized to actinin band and expressed as percentage with respect to control values.

### 2.7 Statistical analysis

Baseline characteristics were analysed by descriptive statistics. Continuous variables were reported as mean ± standard deviation (SD). After assessing for normality, between‐group comparisons were assessed by Mann–Whitney test and Kruskal–Wallis test, as appropriate. The null hypothesis was rejected at *p* < 0.05. To assess the association between variables, Pearson's and Spearman's correlation coefficients were used. Receiver operating characteristics (ROC) curves were generated to evaluate the diagnostic value of serum miRNA in BMD patients compared with healthy controls. Youden's index was used to calculate the best cut‐off values. Statistical analyses were performed with jamovi Version 2.0 and GraphPad Prism Version 9.3.1.

## 3 RESULTS

### 3.1 Characterization of patient cohort

**FIGURE 1:**
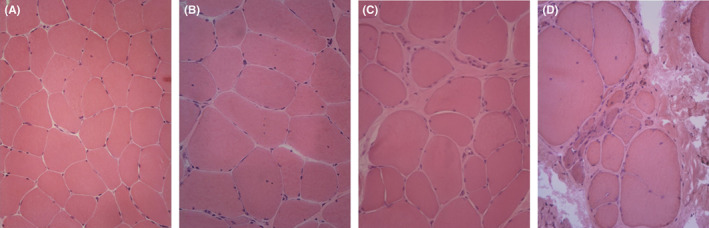
Muscle biopsy. Representative H&E pictures of the normal muscle (A) and in BMD patients. (B) Mild cases showed limited alterations in fibre size, a mild fibrosis, and a few central nuclei. (C) Moderate cases showed an increase in fibre size variability and in fibrotic tissue. (D) Severe cases showed an important presence of fibroadipose tissue, a significant variation in fibre size, with both highly atrophic and hypertrophic fibres and an increase in nuclear centralization

We enrolled 40 individuals, including 29 patients affected with BMD and 11 healthy controls. Mean age at evaluation was 38 ± 11.8 years (range 19–60) in BMD patients and 41.1 ± 13 years (range 24–61) in healthy controls (*p* = 0.469). Mean disease duration in BMD patients was 21.9 ± 8.9 years. Demographic and clinical features of BMD patients are listed in Table 1. Two out of 29 patients were on chronic oral steroid therapy. The histopathological features observed in our cohort of BMD patients showed a marked histological variability ranging from an almost normal morphology to a severe dystrophic pattern. Inflammatory infiltrates were poor or completely absent. The evaluation of MFA and the amount of fibroadipose tissue allowed to divide the patients into three groups. A mild group of 12 patients with a MFA > 80%, a moderate group of 9 patients with intermediate MFA (79%–65%) and a severe group of eight patients with a MFA < 65%. Histological features of the three groups are represented in Figure 1. Neurological assessment evaluated with functional tests (MFM scale, 6MWT and temporized test – 4‐stairs climb, 10 meters and Gowers' time) were significantly different among the three groups, showing worse score in patients with higher histological alterations at muscle biopsy.

**TABLE 1:** Demographic and clinical features of BMD patients

	BMD patients (*n* = 29)	Histopathological alterations			*p*‐Value
		Mild (*n* = 12)	Moderate (*n* = 9)	Severe (*n* = 8)	
Age at evaluation	38 ± 11.8	33.3 ± 10.2	43.6 ± 11.4	38.9 ± 12.9	0.141
Age at onset	16.4 ± 10.0	14.8 ± 7.9	20.7 ± 10.4	14.3 ± 11.9	0.364
Disease duration	21.9 ± 8.9	18.6 ± 7.0	22.9 ± 9.1	25.6 ± 10.5	0.211
EF (%)	60.1 ± 5.1	61.1 ± 3.1	60.6 ± 5.8	58.1 ± 6.7	0.439
CK (U/L)	1739 ± 1528	1486 ± 806	1921 ± 2124	1873 ± 1688	0.927
Dys expression	36.5 ± 15.3	34.5 ± 18.9	34.7 ± 10.5	41.6 ± 14.6	0.574
MFM‐D1	19.7 ± 5.3	23.1 ± 4.9	18.7 ± 2.1	15.9 ± 5.2	**0.003**
MFM‐D2	35.4 ± 1.0	35.8 ± 0.5	35.7 ± 0.5	34.5 ± 1.5	**0.007**
MFM‐D3	20.7 ± 0.5	21 ± 0	20.7 ± 0.5	20.4 ± 0.7	**0.030**
MDM total	75.7 ± 6.2	79.8 ± 5.0	74.9 ± 2.6	70.5 ± 6.6	**0.001**
6MWT	373 ± 73.2	422 ± 59.7	367 ± 49.1	307 ± 63	**<0.001**
4‐Stairs climb (s)	9.9 ± 17.3	5.2 ± 3.2	7.8 ± 7.1	20.8 ± 32.8	**0.011**
10 meters (s)	9.2 ± 6.0	6.9 ± 1.5	11.3 ± 10.1	10.2 ± 2.6	**0.002**
Gowers (s)	16.5 ± 34.7	5.5 ± 2.6	9.2 ± 4.6	45.2 ± 64.6	**0.007**

Abbreviations: 6MWT, 6‐minute walking test; CK, creatine kinase; EF, ejection fraction; MFM, motor function measurement (D1, D2, D3, total).

### 3.2 Serum myomirs are reliable diagnostic biomarkers in BMD patients

**FIGURE 2:**
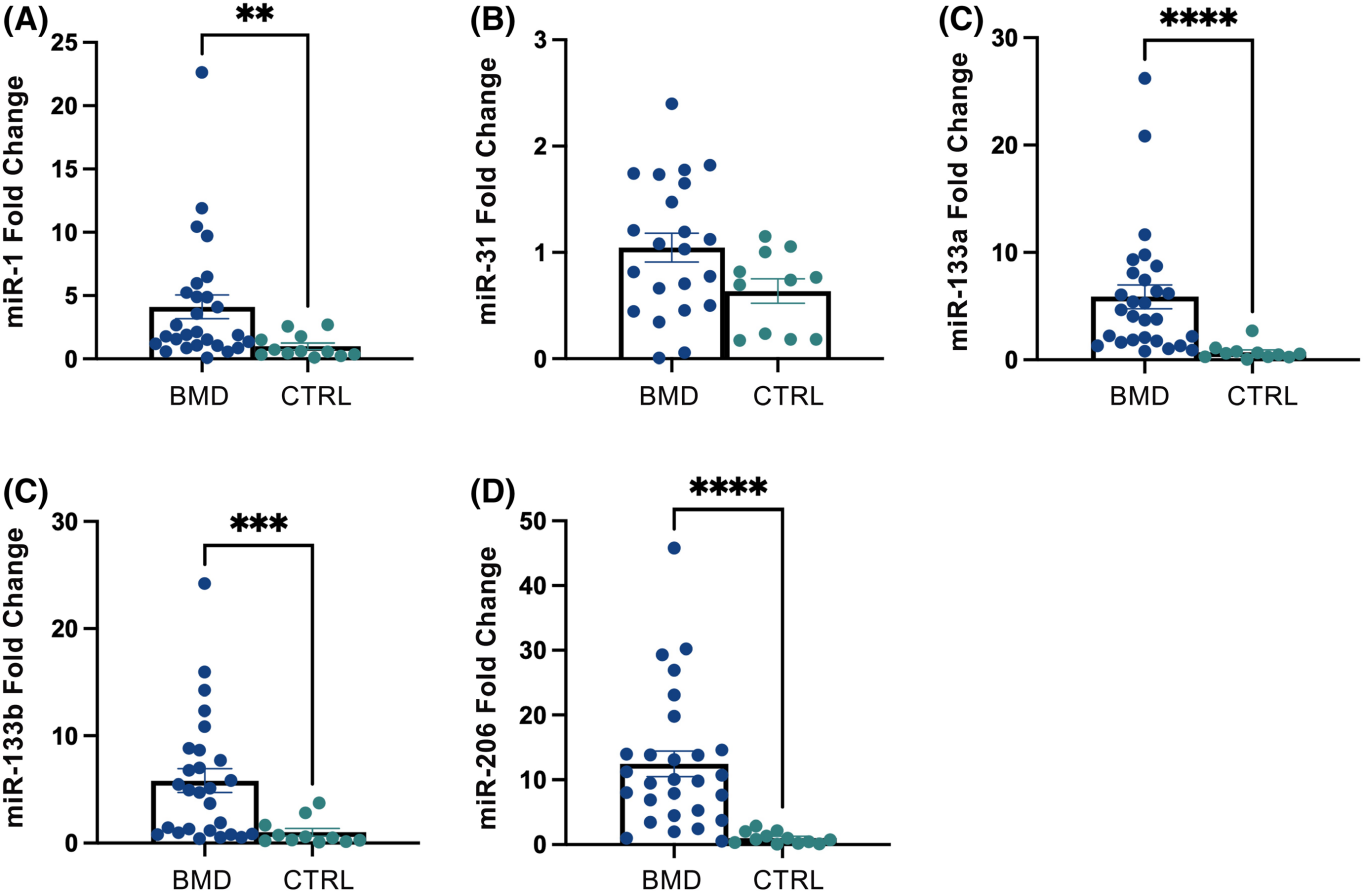
Distribution of serum miRNA in BMD patients and healthy controls. (A) Serum miR‐1 levels were higher in BMD than in healthy controls (*p* = 0.0024). Scatter dot plot values represent means and standard error of the mean (SEM). (B) Serum miR‐31 levels did not significantly differ between BMD and controls (*p* = 0.0822). (C) Serum miR‐133a levels were higher in BMD than in healthy controls (*p* < 0.0001). (D) Serum miR‐133b levels were higher in BMD than in healthy controls (*p* = 0.0002). (E) Serum miR‐206 levels were higher in BMD than in healthy controls (*p* < 0.0001)

**FIGURE 3:**
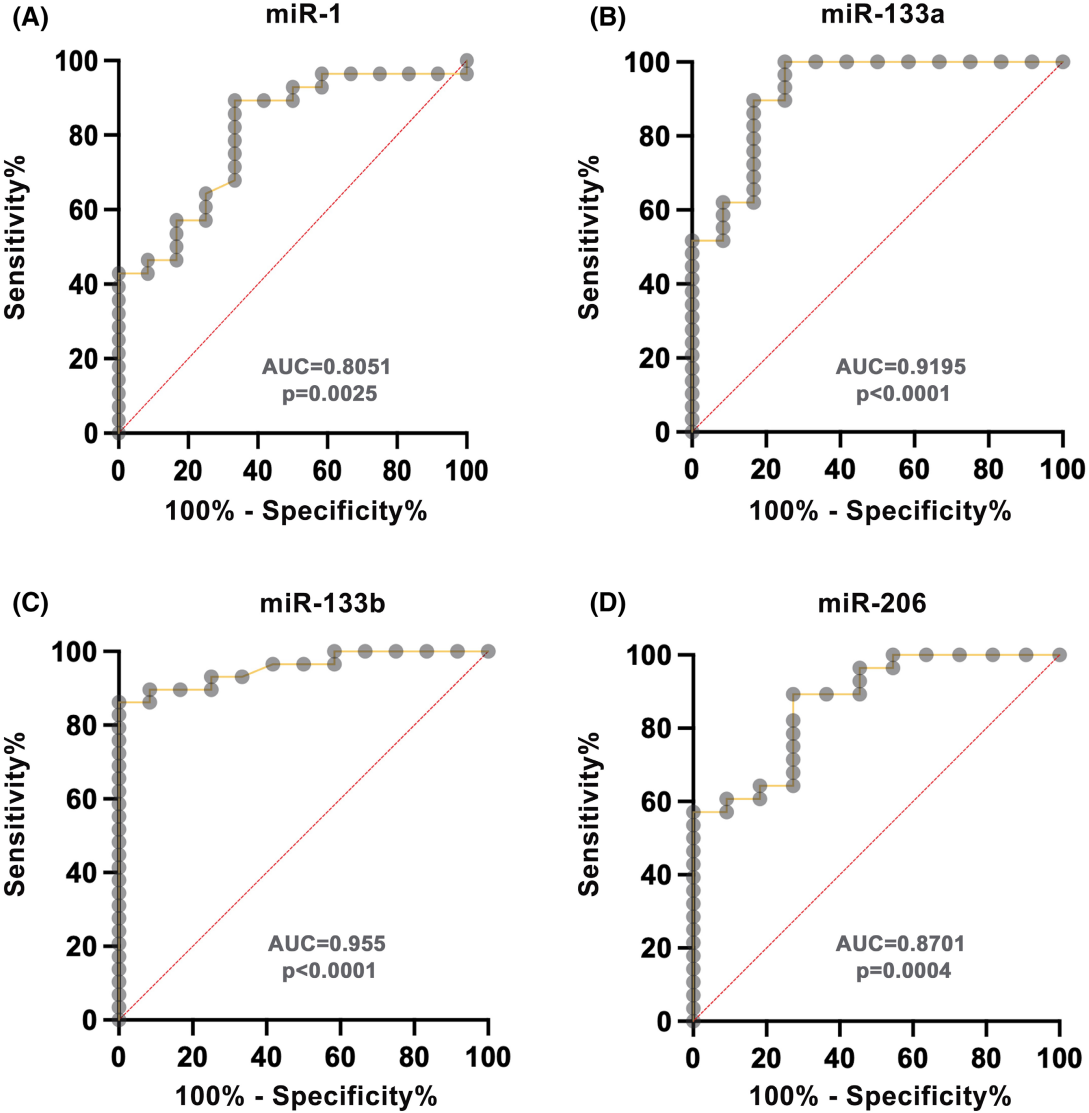
ROC analysis. Accuracy of serum miR‐1 (A), miR‐133a (B), miR‐133b (C) and miR‐206 (D) for the diagnosis of BMD vs healthy controls

Serum levels of the five myomirs were compared between BMD patients and healthy controls. A statistically significant overexpression of serum levels of miR‐1 (*p* = 0.0024), miR‐133a (*p* < 0.0001), miR‐133b (*p* = 0.0002) and miR‐206 (*p* < 0.0001) was found in BMD subjects compared to healthy controls (Figure 2). Serum concentrations of miR‐31 were not significantly different in patients compared to controls (*p* = 0.0822). No difference in serum myomir levels were detected among the three groups of BMD patients (data not shown). ROC analysis was employed to assess whether overexpressed myomirs were able to discriminate BMD patients from healthy controls. All the four myomirs displayed high accuracy for BMD diagnosis when compared to controls (miR‐1 area under the curve [AUC] 0.8051, *p* = 0.0025; miR‐133a AUC 0.9195, *p* < 0.0001; miR‐133b AUC 0.955, *p* < 0.0001; miR‐206 AUC 0.8701, *p* = 0.0004) (Figure 3). Serum cut‐off values better discriminating BMD from healthy controls were 0.0008 for miR‐1 (sensitivity 89.3%, specificity 66.7%), 0.001 for miR‐133a (sensitivity 89.7%, specificity 83.3%), 0.00007 for miR‐206 (sensitivity 89.3%, specificity 72.7%). Serum levels of miR‐133b higher than 0.0007 were the best predictors of BMD diagnosis (sensitivity 86.2%, specificity 100%).

### 3.3 Serum miR‐133b correlates with functional measures and fibrosis in BMD patients

To assess whether myomirs reflected the severity of the disease in patients with BMD, we performed a correlation analysis among serum myomirs in BMD patients and clinical, functional and histological parameters. Serum miR‐133b levels were inversely correlated with scores at MFM D1 (*r* = −0.414, *p* = 0.028) and MFM total (*r* = −0.468, *p* = 0.012). Serum miR‐133b levels were significantly correlated with outcomes obtained in the other functional tests (expressed as time in seconds required to complete the tasks): 4‐stairs climb (sec): *r* = 0.615, *p* < 0.001; Gowers (sec): *r* = 0.685, *p* < 0.001. Serum levels of miR‐1, miR‐133a and miR‐206 were positively correlated with CK levels (*r* = 0.402, *p* = 0.048; *r* = 0.423, *p* = 0.032; *r* = 0.610, *p* = 0.001).

**FIGURE 4:**
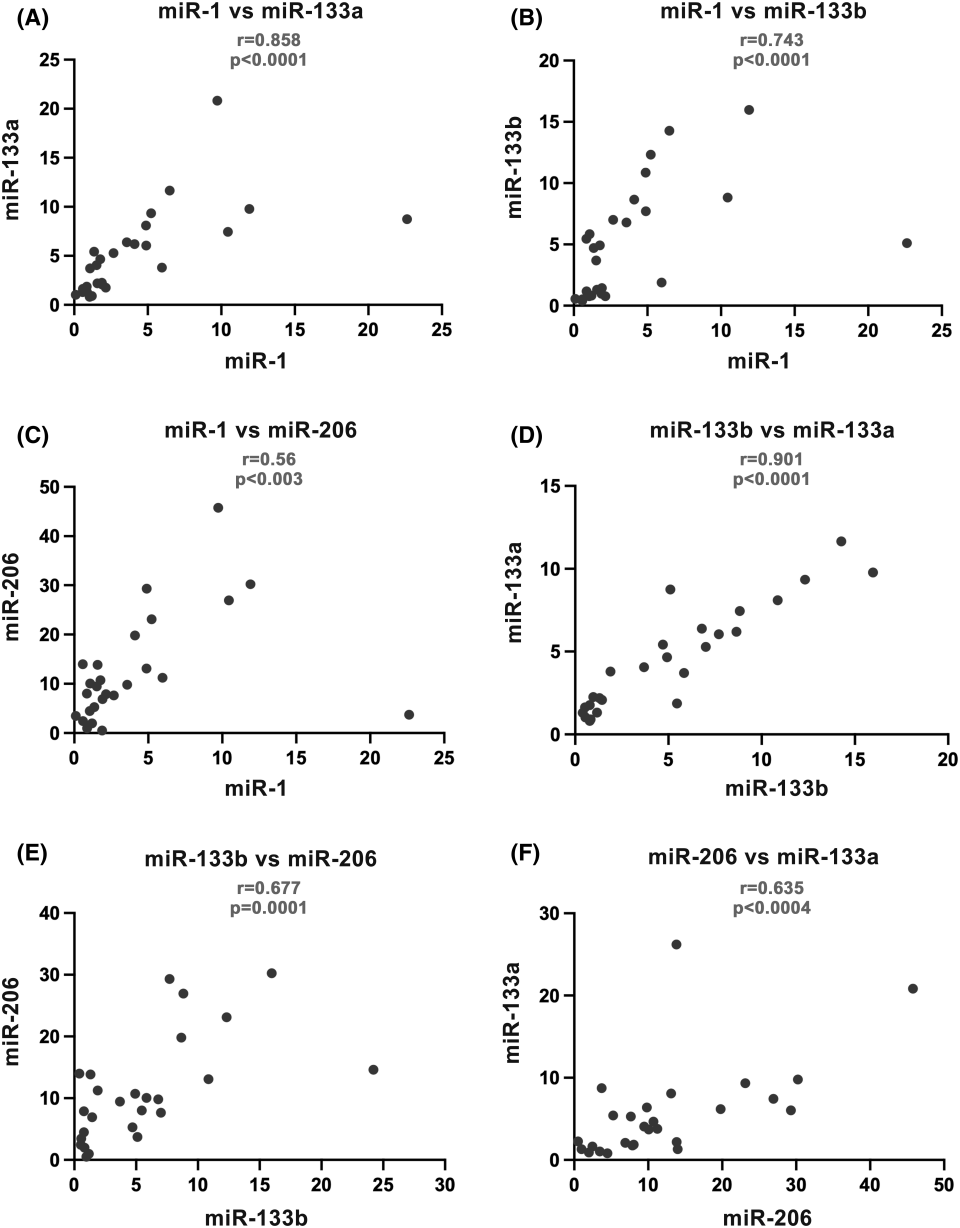
Correlation analysis. (A) miR‐1 and miR‐133a were positively correlated (*r* = 0.858, *p* < 0.0001). (B) miR‐1 and miR‐133b were positively correlated (*r* = 0.743, *p* < 0.0001). (C) miR‐1 and miR‐206 were positively correlated (*r* = 0.56, *p* < 0.003). (D) miR‐133b and miR‐133a were positively correlated (*r* = 0.901, *p* < 0.0001). (E) miR‐133b and miR‐206 were positively correlated (*r* = 0.677, *p* = 0.0001). (F) miR‐206 and miR‐133a were positively correlated (*r* = 0.635, *p* < 0.0004). Spearman's correlation coefficient was used

Serum miR‐133b was not significantly associated with MFA (*r* = −0.293, *p* = 0.13) but was significantly correlated with increased fibrosis at muscle biopsy (*r* = 0.387, *p* = 0.042). No significant correlation between the other myomirs and clinical, functional and histological parameters were found, including dystrophin expression. Further, we did not find any significant correlation between the grade of fibrosis and disease duration. Finally, aside from miR‐31, serum levels of myomirs (miR‐1, miR‐133a, miR‐133b and miR‐206) were positively correlated among them in BMD population (Figure 4).

## 4 DISCUSSION

Becker muscular dystrophy is characterized by high clinical and histological heterogeneity, which reflect the high variability of the pathological process underlying this disease. The identification of specific miRNAs in serum of BMD patients may add a piece of knowledge on pathogenic mechanisms and provide new tools for patient stratification and prognosis prediction. At first, serum myomirs were considered related to passive release from damaged myofibers or sarcolemmal dysfunction. Actually, the concordance between muscle and serum levels of these miRNAs is not always present: dystrophic muscles presented reduced levels of miR‐1 and normal levels of miR‐206, miR‐133a and miR‐133b compared to healthy controls.^29^ Therefore, more complex mechanisms have been suggested, including abnormal excretion due to dystrophin deficiency or selective release during muscle differentiation and regeneration.^30^ Moreover, since new treatments are emerging, there is an urgent need for measurable and non‐invasive indicators of therapeutic response.

In this work, we have highlighted the importance of serum miRNAs as potential diagnostic and prognostic biomarkers for BMD. We found that 4 out of 5 myomirs (miR‐1, mir‐133a, miR‐133b and miR‐206) are significantly overexpressed in BMD patients compared with healthy controls. We identified serum miRNA cutoffs which accurately discriminate dystrophic patients from controls, confirming the involvement of these miRNAs in the pathophysiology of BMD. Although miR‐133b is correlated with the degree of fibrosis, we did not find significant variations of myomir levels within the three groups of patients according to the severity of histologic alterations (fibrosis and fibre area) detected at muscle biopsy. This could depend on the features of our patient cohort, which includes ambulant patients (able to walk between 200 and 450 meters at 6MWT) and without a severe cardiomyopathy and which does not encompass asymptomatic or mild symptomatic patients, as well as patients with more severe muscle impairment. However, serum levels of miR‐133b are associated with patients' motor performances (MFM D1, MFM total, 4‐stairs‐climb and Gowers), suggesting that miR‐133b might be a useful prognostic tool for BMD patients.

Most of the studies on myomirs have been performed on DMD patients, in which serum levels of miR‐1, miR‐133a, miR‐133b and miR‐206 are significantly increased.^27^, ^31^, ^32^, ^33^, ^34^ Conversely, data on miR‐31 are conflicting, since they have been found mildly elevated by Zaharieva et al.^29^ and reduced by Vigner group.^34^ Myomirs serum levels in ambulant DMD patients were inversely correlated with muscle strength and increased with lower scores at MRC scale, temporized tests and NSAA scale.^31^, ^32^ Considering a more heterogeneous cohort of patients, Zaharieva et al. evidenced higher levels of dystromyrs (miR‐1, miR‐31, miR‐206, miR‐133a, miR‐133b) in ambulant patients compared to non‐ambulant ones.

A mild serum overexpression of miR‐206, as well as an increase in miR‐1 and miR‐133, has been reported also in BMD patients by Cacchiarelli et al.^31^ Other studies investigated serum levels of myomirs in dystrophic patients, identifying intermediate concentrations in BMD patients in respect with DMD patients and healthy controls.^28^, ^30^, ^34^, ^36^ Upregulation of serum miR‐206 has been found in BMD patients compared to controls, without differences between patients receiving corticosteroid treatments and untreated ones, while miR‐1 and miR‐133a/b concentrations were variable in BMD cohort.^36^

Dysregulation of these dystromirs and a subsequent normalization after morpholino‐mediated dystrophin restoration or exon 23 skipping through adeno‐associated virus or morpholino administration has been demonstrated also in muscle of *mdx* mice.^31^, ^37^, ^38^, ^39^ Twelve‐week treatment with antisense oligomers inducing exon 51 skipping (Eteplirsen) modulated miRNA concentrations in serum of DMD patients.^29^

Our findings are consistent with literature data and confirm the utility of serum myomirs as biomarkers for patient classification and prognosis in BMD. In addition, it gives a detailed characterization of a broader cohort of BMD patients and provides defined cut‐off values of serum myomirs able to discriminate patients from healthy controls. Further studies on a bigger scale, including both asymptomatic and severe BMD patients, could be performed to evaluate how serum levels of myomirs behave in these populations. The combination of serum miRNA quantification with other indicators of disease severity and progression, such as functional tests, muscle MRI and quantification of fibrosis at muscle biopsy, may increase patient stratification for clinical trial enrollment and provide a more precise appraisal of response to pharmacologic treatments.

## AUTHOR CONTRIBUTIONS

**Delia Gagliardi:** Investigation (equal); methodology (equal); visualization (equal); writing – original draft (equal). **Mafalda Rizzuti:** Investigation (equal); methodology (equal); visualization (equal); writing – original draft (equal). **Roberta Brusa:** Conceptualization (equal); investigation (equal); writing – original draft (equal). **Michela Ripolone:** Investigation (equal); methodology (equal). **Simona Zanotti:** Investigation (equal); methodology (equal). **Elisa Minuti:** Investigation (equal). **Valeria Parente:** Investigation (equal); methodology (equal). **Laura Dioni:** Methodology (equal). **Sara Cazzaniga:** Conceptualization (equal); supervision (equal). **Paolo Bettica:** Conceptualization (equal); supervision (equal). **Nereo Bresolin:** Supervision (equal). **Giacomo Pietro Comi:** Conceptualization (equal); supervision (equal); writing – review and editing (equal). **Stefania Corti:** Conceptualization (equal); supervision (equal); writing – review and editing (equal). **Francesca Magri:** Conceptualization (equal); data curation (equal); supervision (equal). **Daniele Velardo:** Conceptualization (equal); data curation (equal); supervision (equal).

## CONFLICT OF INTEREST

The authors declare no competing interests.

## Data Availability

The data that support the findings of this study are available from the corresponding author upon reasonable request.
